# *SlMYC2* mediates stomatal movement in response to drought stress by repressing *SlCHS1* expression

**DOI:** 10.3389/fpls.2022.952758

**Published:** 2022-07-22

**Authors:** Bing-Qin Xu, Jing-Jing Wang, Yi Peng, Huang Huang, Lu-Lu Sun, Rui Yang, Lin-Na Suo, Shao-Hui Wang, Wen-Chao Zhao

**Affiliations:** ^1^College of Plant Science and Technology, Beijing University of Agriculture, Beijing, China; ^2^Bei Jing Bei Nong Enterprise Management Co., Ltd., Beijing, China; ^3^Beijing Key Laboratory for Agricultural Application and New Technique, Beijing University of Agriculture, Beijing, China; ^4^Beijing Academy of Agricultural and Forestry Sciences, Beijing, China

**Keywords:** MYC2 transcription factor, guard cells, abscisic acid, jasmonates, ROS, *SlCHS1*

## Abstract

Drought stress limits plant development and reproduction. Multiple mechanisms in plants are activated to respond to stress. The MYC2 transcription factor is a core regulator of the jasmonate (JA) pathway and plays a vital role in the crosstalk between abscisic acid (ABA) and JA. In this study, we found that *SlMYC2* responded to drought stress and regulated stomatal aperture in tomato (*Solanum lycopersicum*). Overexpression of *SlMYC2* repressed *SlCHS1* expression and decreased the flavonol content, increased the reactive oxygen species (ROS) content in guard cells and promoted the accumulation of JA and ABA in leaves. Additionally, silencing the *SlCHS1* gene produced a phenotype that was similar to that of the *MYC2-*overexpressing (*MYC2*-OE) strain, especially in terms of stomatal dynamics and ROS levels. Finally, we confirmed that *SlMYC2* directly repressed the expression of *SlCHS1*. Our study revealed that *SlMYC2* drove stomatal closure by modulating the accumulation of flavonol and the JA and ABA contents, helping us decipher the mechanism of stomatal movement under drought stress.

## Introduction

The stomatal pore, which is surrounded by two guard cells, is vital for facilitating gas exchange and water by regulating the stomatal aperture, and the method of stomatal action depends on the integration of signals inside and outside the organism (Joshi-Saha et al., 2011). Many environmental stimuli, such as CO_2_ fluctuation, various stresses, and multiple phytohormones, modulate stomatal movements. Water loss might cause plants to become dehydrated in the presence of excessive drought or high temperatures, and the mechanism controlling the size of the stomatal aperture *via* dynamic adjustment of the turgor of guard cells is essential to prevent excess water loss (Kim et al., 2017). Ion exchange, metabolites, and the regulation of gene expression in guard cells have been shown to drive stomatal aperture modulation (Hirayama and Shinozaki, 2007).

Abscisic acid (ABA), a core hormone, cooperates with jasmonates (JAs), auxins, cytokinins, and ethylene to regulate stomatal movements (Nemhauser et al., 2006; Huang et al., 2008). As a part of the water loss response, ABA interacts with reactive oxygen species (ROS) to stimulate stomatal closure. ROS are secondary messengers that are involved in guard cell signalling pathways (Zhang et al., 2001; Mittler et al., 2011; Munné-Bosch et al., 2013). For example, ROS bursts are presumed to target calcium channels and promote the influx of calcium across the plasma membrane and the release of calcium from internal organelles in guard cells, inducing the activation of anion efflux channels and triggering stomatal closure (Cho et al., 2009; Wang et al., 2013). Moreover, ROS levels are associated with cellular redox networks *via* glutaredoxins, peroxiredoxins, thioredoxins, and/or nicotinamide adenine dinucleotide phosphate (NADPH, Moon et al., 2003; Dietz et al., 2010; Rouhier, 2010), and the ROS level is strictly regulated by the production of various enzymes and small-molecule antioxidants, such as ascorbic acid, glutathione, and flavonoids, to maintain homeostasis (Mittler et al., 2004; Singh et al., 2016; Zandalinas et al., 2016).

JAs are signalling molecules that regulate stress responses and developmental processes (Huang et al., 2017; Per et al., 2018). During drought stress, JA synergistically activates stress responses with ABA to regulate stomatal closure (Munemasa et al., 2007; Kim et al., 2017; Raza et al., 2021). Stress-induced JA and ABA production mediates stomatal closure by activating extracellular Ca^2+^ influx and/or H_2_O_2_/NO signalling (Harrison, 2012). The generation of ROS and NO in guard cells is induced by both ABA and JA (Munemasa et al., 2007). The crosstalk between JA and ABA is mainly reflected in the fact that they both induce *MYC2* expression (Anderson et al., 2004; Jensen et al., 2010; Hossain et al., 2011). MYC2 is a hub of JA signalling that transactivates various JA-responsive genes in response to mechanical damage, oxidative stress, pathogens, and drought stress in some plants (Dombrecht et al., 2007; Kazan and Manners, 2008). *AtMYC2* in *Arabidopsis* was first reported to be induced by ABA treatment and drought conditions (Abe et al., 1997), and this transcription factor was originally described as an activator of the ABA signalling pathway in plants under drought stress (Abe et al., 2003). The *AtMYC2* homologue in rice (*OsbHLH148*) is involved in ABA- and JA-dependent responses to drought stress (Seo et al., 2011). However, the mechanism of the *MYC2*-mediated drought response remains unclear.

Flavonoids are the largest family of polyphenols and exhibit a wide range of biological activities, protecting plants from different biotic and abiotic stresses, such as heat and drought stress (Peters and Constabel, 2002; Schmidt et al., 2012; Tohge et al., 2013; Shen et al., 2019). The first step of flavonoid biosynthesis is catalysed by chalcone synthase (CHS, Liu et al., 2021). Flavonol metabolites, as antioxidants, regulate ROS-modulated root growth in tomato and guard cell signalling in *Arabidopsis* (Maloney et al., 2014; Watkins et al., 2014; An et al., 2016). Watkins et al. (2014) found that flavonols specifically accumulate in guard cells, and *atchs* null mutants exhibit more rapid ABA-induced closure and increased ROS contents in guard cells in *Arabidopsis*. A similar phenomenon was observed in tomato, in which flavonols inhibit the ROS burst that activates stomatal closure (Watkins et al., 2017).

Flavonoid biosynthesis is transcriptionally regulated by R2R3-MYB proteins, which control flavonol biosynthesis by activating early biosynthetic steps, and the MYB-bHLH-WD40 complex, which activates late biosynthetic genes (Li, 2014). According to a study by Ogawa et al. (2017), the expression of genes downstream of flavonoid biosynthesis, including chalcone synthase (*CHS*), chalcone isomerase (*CHI*), and acyl-CoA synthetase (*ACS*), is induced by JA through *OsMYC2* in rice. In contrast, in *Arabidopsis*, the expression of *CHS* genes is markedly increased in the *atmyc2*-3 mutant compared to its wild-type, suggesting that *AtMYC2* negatively regulates the expression of *CHS* genes upon exposure to blue light (Yadav et al., 2005). Based on different regulatory patterns in different scenarios, the regulatory relationship between *MYC2* and *CHS* during the drought response in plants is worth clarifying.

Tomato *SlMYC2* is involved in the drought stress response. *SlMYC2*-overexpressing plants were adopted to dissect the potential mechanism of the *SlMYC2*-mediated drought stress response, and *SlMYC2* overexpression led to increased ROS accumulation in guard cells, decreased stomatal aperture and increased drought tolerance. Further investigations showed that *SlMYC2* negatively regulated flavonol synthesis by directly interacting with the *SlCHS1* promoter and positively modulated the JA and ABA contents. Finally, silencing the *SlCHS1* gene led to a decrease in flavonol contents and an increase in ROS levels in guard cells, which promoted stomatal closure in tomato.

## Materials and methods

### Plant growth and drought treatment

The wild-type tomato (*Solanum lycopersicum*) cv. Castlemart (CM) and M82 were used in the present study. *SlMYC2*-overexpressing transgenic plants (*MYC2*-OE, M82 genetic background) were kindly provided by the State Key Laboratory of Plant Genomics of the Chinese Academy of Sciences (Beijing, China). The tomato seeds were germinated for 48 h in the dark at 26°C until radicles were visible on moistened filter paper in Petri dishes (8.5-cm diameter). Subsequently, they were sown in plug trays (27-cm width, 54-cm length, 50 holes) containing a mixture of 50% (v/v) vermiculite and 50% (v/v) peat soil in a semicontrolled greenhouse with a photoperiod of 16 h, 26°C/8 h, 20°C day/night regime (with a white light intensity of 200 μmol m^−2^ s^−1^), and 80% relative humidity. When seedlings had developed two true leaves (14 days after sowing seeds), the uniformly sized seedlings were transplanted into pots (10-cm diameter, 8.5-cm height, one plant per pot) with the same substrate.

For the water loss experiment, the 28-day-old seedlings were divided into well-watered (mock) and water loss (stress) groups, with 10 plants per group, and arranged in a randomized block design: (1) mock, 26/20°C with irrigation with a definite amount of water every day to maintain 40% soil volumetric water content; and (2) water loss, 26/20°C without irrigation. The pots were covered with foil to prevent soil water loss. Leaves were collected for subsequent analyses. The recovery phenotype was documented at 3 h after rewatering.

### Isolation of the full-length tomato *CHS1* cDNA and construction of TRV plasmids

An approximately 300 bp fragment of the *SlCHS1* (Solyc09g091510) cDNA was cloned into the vector pTRV2 and then transformed into *Agrobacterium tumefaciens* GV3101 (Weidi, Shanghai, China). The TRV2-based VIGS system was conducted by sprout vacuum infiltration using the method reported by Yan et al. (2012).

### Measurement of the RWC and biomass

For the determination of the RWC, leaves from six plants per accession cultivated under mock and stress conditions were collected. Fresh weight (FW) was determined immediately after cutting the leaves from the plants. The saturated weight (SW) of leaves was determined after 12 h of water absorption. The dry weight (DW) was determined after 6 h of incubation at 70°C. The leaf RWC was calculated as (FW − DW)/(SW − DW) × 100.

Six plants per accession of each treatment were randomly selected to measure the biomass using the drying method. The aboveground parts were cut off, and the dry weight was measured as the shoot biomass (W_S_). Then, the roots with soil were washed with water, and the dry weight was used as the root biomass (W_R_). The biomass of the whole plant was calculated as W_S_ + W_R._

### Quantitative real-time PCR

The 5th true leaves were harvested starting from the apex of three plants under well-watered and water loss conditions. For qPCR of leaves, samples were pooled and ground in liquid nitrogen in triplicate to obtain three biological replicates. For qPCR of guard cells, the isolation of guard cell protoplasts from epidermal fragments of tomato leaves was conducted using the method developed by Yao et al. (2018).

RNA extraction and quantitative real-time PCR were conducted using the HiPure Plant RNA Mini Kit (Magen, Shanghai, China) and the ReverTra Ace™ qPCR Kit (TOYOBO, Osaka, Japan) according to the manufacturers’ instructions. The coding sequences of internal references and genes to be tested were searched on the Phytozome and Sol Genomics Network websites. All primers are listed in Supplementary Table S1. qPCR was performed in triplicate using 5 ng of cDNA, 5 μl of SYBR Green qPCR Master Mix (TOYOBO, Osaka, Japan), and 0.4 μM of the primers listed in Supplementary Table S1. The relative expression levels were calculated using the 2^−ΔΔCT^ method (Livak and Schmittgen, 2001) with tomato *Actin2* as an internal reference.

### Measurement of the stomatal aperture

The epidermal peels were collected from plants after 8 days of water loss treatment and from well-watered plants and then fixed with absolute alcohol for 3 min. Images of stomata were observed using a B302 Series Biological Microscope (Optec, Chongqing, China). The length and width of the stomatal aperture were determined using PhotoRuler software. The stomatal aperture was calculated by determining the width:length ratio. The percentage of open stomata was determined by dividing the number of open stomata by the total number of stomata.

### Measurement of ABA and JA contents

Leaves of *MYC2*-OE and M82 plants were harvested at 0, 4 and 8 days of water loss or well-watered conditions for ABA and JA measurements. ABA and JA were extracted using an isopropyl alcohol/water/hydrochloric acid extraction method, and the ABA and JA levels were quantified using LC–MS (Agilent 1260 Infinity-Agilent 6420A) with an internal standard as previously described (Forcat et al., 2008; Izumi et al., 2009). Three replicates were measured for each data point.

### Quantification of flavonol contents using DPBA staining

Leaves were cut off and immersed in a solution containing 2.52 mg/ml DPBA and 0.01% (v/v) Triton X-100 for 2 h and then washed with distilled water for 2 min, which was repeated three times. Then, the abaxial leaf epidermis was torn off, transferred to a microscope slide, and mounted in deionized water between two coverslips. A Leica TCS SP5 (Leica, Germany) laser-scanning confocal microscope was used for exciting the blade surfaces with 100% laser power at a wavelength of 488 nm. The optimized LSCM setup and quantification of fluorescence were performed according to previous research to differentiate between DPBA and chlorophyll fluorescence (Watkins et al., 2014). Fluorescence was quantified in each guard cell using Image-Pro Plus software (United States).

### Quantification of ROS contents in guard cells

The ROS content in guard cells was tested using the H_2_DCFDA fluorescent dye (Invitrogen, United States). The epidermal peels of fully expanded tomato leaves from stressed and mock plants were floated on loading buffer containing 10 mM MES-Tris (pH 6.15) and 50 μM H_2_DCFDA for 15 min in the dark and then washed 3 times with 10 mM MES-Tris to remove excess dye from the apoplast. A Leica TCS SP5 (Leica, Germany) LSCM was used to examine the fluorescence with an excitation wavelength of 488 nm. During observation and quantification, all settings and operations were performed as described previous research (Watkins et al., 2014). Fluorescence quantified in each guard cell using Image-Pro Plus software.

### Chromatin immunoprecipitation (ChIP)-qPCR

The ChIP assay was performed using 21-day-old 35S::*MYC2*-GFP (*MYC2-*OE) tomato plant leaves with the EpiQuik™ Plant ChIP Kit (EpiGentek, New York, United States). For the qPCR experiment, *Actin2* was used as the internal reference, and the enrichment of the DNA fragments was quantified. At least three biological replicates were conducted. The primers used here are listed in Supplementary Table S1.

### Yeast one-hybrid assay

Yeast one-hybrid assays were performed using the Matchmaker Gold Yeast One-Hybrid System (Clontech) according to the manufacturer’s instructions. The coding region of *SlMYC2* was inserted into the pGADT7 vector (Clontech, United States) to construct the transcription factor expression vector. The promoter fragment of *SlCHS1* was inserted into the pBait-AbAi vector. The Y1HGold yeast strain containing the pGADT7 recombinant vector was grown on medium lacking Leu for 72 h at 30°C. Then, the interaction between *SlMYC2* and the *SlCHS1* promoter was tested on medium lacking Leu and with AbAi added for 72–120 h at 30°C. The empty pGADT7 vector was used as the control.

### Luciferase reporter assay

The ORFs of *SlMYC2* were inserted into the pGreenII 62-SK effector expression system. The promoter sequence of *SlCHS1* was integrated into the pGreen-0800-LUC vector. Both vectors were transferred into *Agrobacterium rhizogenes*. Equal volumes of the *Agrobacterium* solution containing LUC reporter or effector were mixed and transfected into tobacco leaves. After 48 h, the LUC activity was determined using a Multimode Plate Reader (Victor X4, PerkinElmer). The levels of inhibition or activation were calculated based on the ratio of LUC activity.

### Statistical analysis

**Figure 1:**
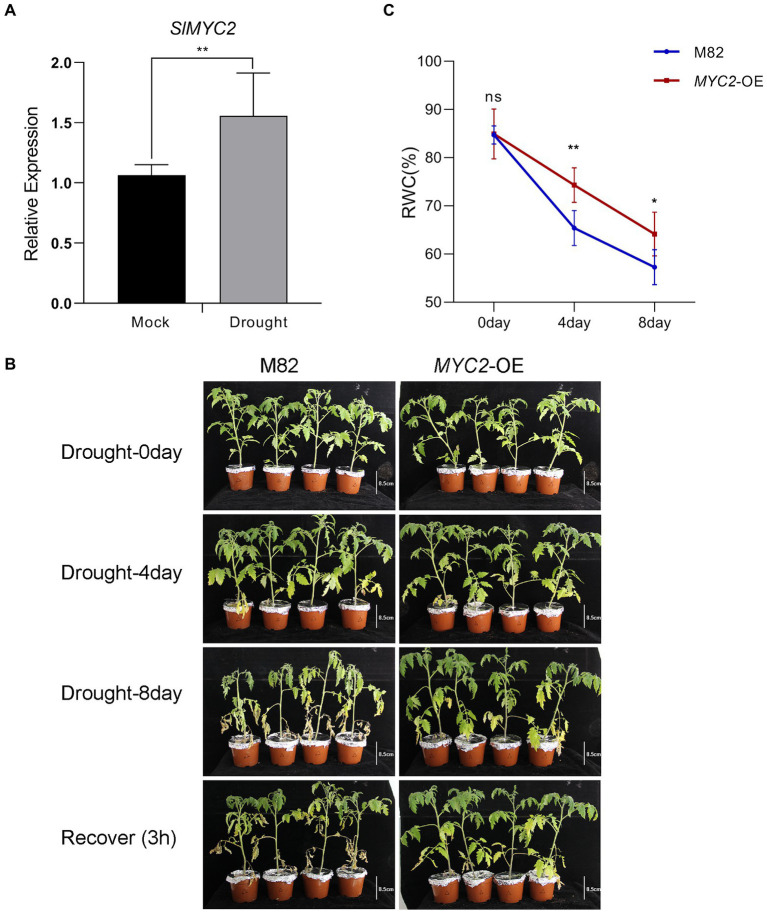
Increased tolerance of *SlMYC2*-overexpressing plants to drought stress. **(A)** *SlMYC2* expression patterns in tomato plant leaves under drought stress compared with mock treatments. **(B)** The phenotypes of *MYC2*-OE and M82 plants under water loss conditions (bar = 8.5 cm). **(C)** RWC of leaves of *MYC2*-OE and M82 plants during natural water loss. Asterisks represent significant differences (*p < 0.05 and **p < 0.01) determined using Student’s *t* test and Tukey’s multiple comparisons test.

**Figure 2:**
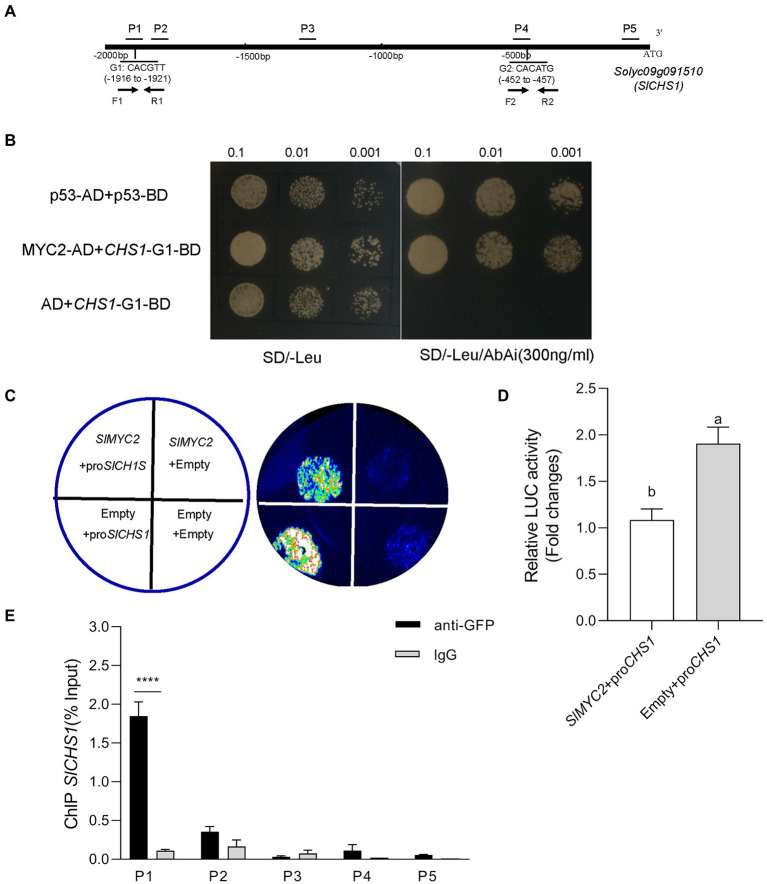
SlMYC2 directly binds to the *SlCHS1* promoter. **(A)** The distribution of fragments of the *SlCHS1* promoter (P1–P5) used in ChIP–qPCR assays and two G-box motifs tested in the Y1H assay (G1 and G2). **(B)** Interaction of SlMYC2 with the *SlCHS1* promoter fragments in the Y1H assay. **(C,D)** Effects of SlMYC2 on the *SlCHS1* promoter in the dual-luciferase assay. **(E)** ChIP–qPCR was used to analyse the recruitment of SlMYC2 to the *SlCHS1* promoter region. Different letters indicate significant differences (*p* < 0.01) determined using Student’s *t* test. Asterisks represent significant differences (****p < 0.0001) determined using Tukey’s multiple comparisons test.

Statistical analyses of the data were performed using GraphPad Prism software. The significance of differences in *SlMYC2* expression presented in Figure 1A and relative LUC activity in Figure 2D were assessed using Student’s *t* test (*n* = 6). The stomatal aperture, the percentage of open stomata, and the DCF and DPBA fluorescence of each accession under each water condition were analysed using one-way ANOVA, followed by Tukey’s multiple comparisons test (*n* = 500). For each accession, RWC (*n* = 6) and ABA and JA synthesis-related gene expression (*n* = 3) were analysed using two-way ANOVA with duration and water conditions as the main factors, and *post hoc* comparisons between treatments for the same accession were performed using Tukey’s test. Data are presented as the means ± standard errors.

## Results

### *SlMYC2* responds to drought stress in tomato

We adopted quantitative PCR (qPCR) to assess the pattern of *SlMYC2* expression in M82 at the four leaf stage under drought stress to investigate whether *SlMYC2* was implicated in the drought stress response. The results showed that *SlMYC2* expression was obviously induced by drought compared to the mock treatment (Figure 1A), suggesting that *MYC2* was involved in the drought response.

**Figure 3:**
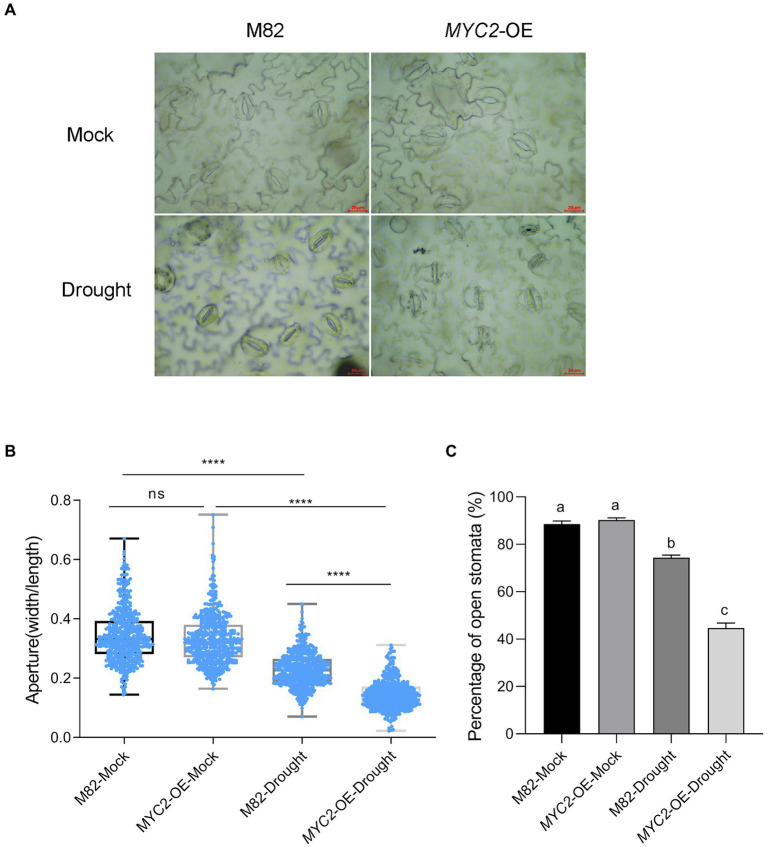
Overexpression of *SlMYC2* promoted stomatal closure in plants under drought stress. **(A)** Typical images of stomata of *MYC2*-OE and M82 plants after 8 days of water loss. **(B)** Stomatal aperture of *MYC2*-OE and M82 plants with and without 8 days of water loss (M82: n = 506; *MYC2*-OE: *n* = 501; M82-Drought: *n* = 504; *MYC2*-OE-Drought: *n* = 501). Data were obtained from three independent experiments (bar = 20 μm). **(C)** The percentage of open stomata in *MYC2*-OE and M82 plants with and without 8 days of water loss (170 for each replicate, three replicates, *n* = 510). Asterisks and different letters represent significant differences (*p* < 0.0001) based on Tukey’s multiple comparisons test.

We conducted a water loss experiment and measured the leaf relative water content (RWC), stomatal aperture and ROS accumulation in the transgenic *SlMYC2*-overexpressing (*MYC2*-OE) plants and wild-type M82 plants to assess the role of *SlMYC2* in drought resistance in tomato. The leaves of *MYC2*-OE and M82 plants were clearly withered and yellow in response to water loss compared with well-watered conditions (Figure 1B and Supplementary Figure 1). The RWC of *MYC2-*OE leaves was significantly higher than that of M82 leaves at 4 days and 8 days during water loss (Figure 1C). The biomass of whole plants and shoots of *MYC2*-OE plants was also determined during water loss, indicating that the biomass of *MYC2*-OE was significantly greater than that of M82 at 8 days ( Supplementary Figure 2). These results supported the positive effect of *SlMYC2* on drought resistance. After 8 days of water loss, images of the epidermal peels of *MYC2-*OE and M82 plants were captured with an optical microscope and used to measure stomatal aperture (Figure 3A). Apertures were quantified for five hundred stomata from three independent experiments. *MYC2-*OE and M82 exhibited similar apertures under mock conditions, and the stomatal apertures of both *MYC2-*OE and M82 plants under water stress conditions were remarkably smaller than those under the mock conditions (Figures 3A,B). Moreover, the stomatal aperture of *MYC2-*OE plants was smaller than that of M82 under drought stress (Figures 3A,B). In addition, a lower percentage of open stomata was observed in *MYC2-*OE plants during drought stress (Figure 3C).

**Figure 4:**
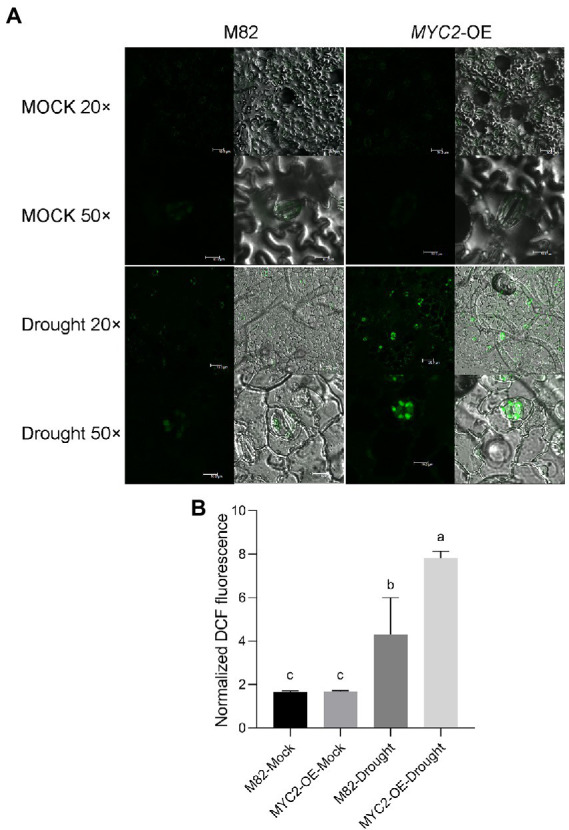
*SlMYC2* overexpression increased ROS accumulation in guard cells under drought stress. **(A)** Confocal microscopic images of H_2_DCF-DA-stained guard cells of *MYC2*-OE and M82 plants from 8-day water loss and mock treatment groups. DCF fluorescence is shown in green (bar = 30 μm for 20×; bars = 10 μm for 50×). **(B)** Quantification of DCF fluorescence in guard cells (M82: *n* = 499; *MYC2*-OE: *n* = 505; M82-Drought: *n* = 505; *MYC2*-OE-Drought: *n* = 509). Data were obtained from three experiments. Different letters represent significant differences (*p* < 0.0001) determined using Tukey’s multiple comparisons test.

ROS bursts are an essential feature of rapid stomatal closure. The ROS concentrations in guard cells of *MYC2-*OE and M82 plants subjected to different irrigation regimes were quantified using the ROS fluorescent probe 2′,7’dihydrodichloro-fluorescein diacetate (H_2_DCF-DA, Halliwell and Whiteman, 2004). We quantified the levels of DCF fluorescence in five hundred guard cells of the wild-type and *MYC2-*OE plants, and the values were normalized to the levels in M82 cells under well-watered conditions. Under the well-watered condition, no difference was observed in ROS contents between the *MYC2-*OE and wild-type plants (Figures 4A,B). After 8 days of water loss, the ROS concentrations in guard cells of all plants were significantly increased, and the extent of the increase in *MYC2-*OE plants was markedly higher than that in M82 plants (Figures 4A,B). Taken together, all these data suggested that *SlMYC2* overexpression resulted in lower sensitivity to drought stress, possibly *via* ROS-mediated stomatal closure.

### *SlMYC2* promoted ABA and JA biosynthesis in plants under drought stress

**Figure 5:**
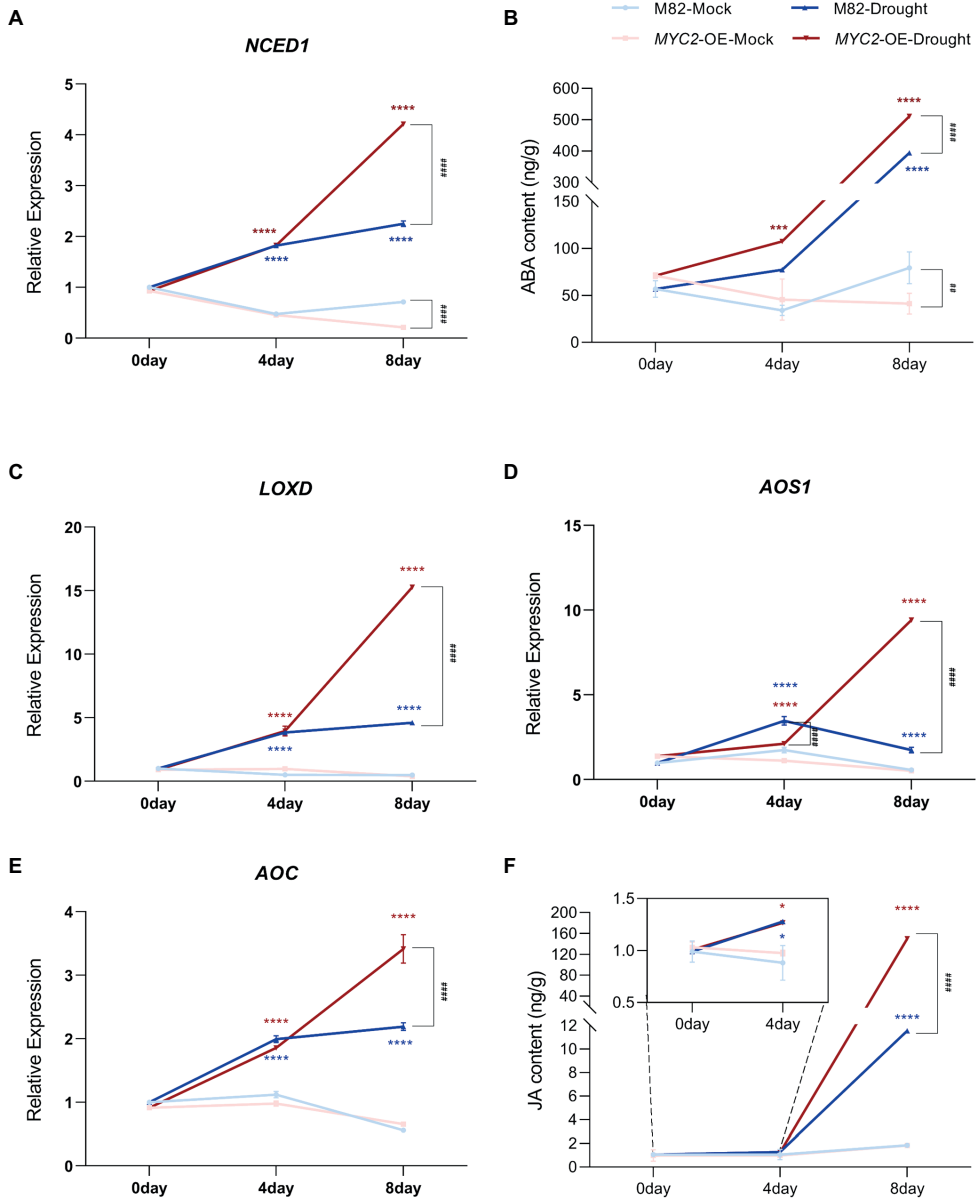
*SlMYC2* promoted ABA and JA biosynthesis in plants under drought stress. **(A,C–E)** *NCED1, LOXD, AOS1* and *AOC* expression patterns in leaves of *MYC2*-OE and M82 plants under drought stress and mock conditions. **(B,F)** The ABA and JA contents in leaves of *MYC2*-OE and M82 plants under drought stress and mock conditions. Asterisks represent significant differences (*p < 0.01, ***p < 0.001, and ****p < 0.0001) between water loss and mock conditions in *MYC2*-OE (red) or M82 (blue) plants; pound signs indicate significant differences (^###^*p* < 0.001 and ^####^*p* < 0.0001) between *MYC2*-OE and M82 plants under the same treatment conditions, as determined using Tukey’s multiple comparisons test.

Previous studies have revealed that both ABA and JA regulate stomatal movement in response to water loss stress, and ABA plays a key role in this process (Munemasa et al., 2007; Kim et al., 2010). We first measured the expression of genes related to ABA and JA synthesis in M82 and *MYC2*-OE subjected to different irrigation regimes to investigate the modulation of JA and ABA levels by *SlMYC2*. The expression of the ABA synthesis gene 9-cis-epoxycarotenoid dioxygenase 1 (*NCED1*) was significantly upregulated in response to drought stress. Although the expression of *NCED1* in *MYC2*-OE was lower than that in M82 after 8 days under well-watered conditions, it was significantly higher than that in M82 under water loss conditions (Figure 5A). Correspondingly, a similar change in the ABA content was also observed (Figure 5B). Meanwhile, the expression levels of the JA synthesis-related genes lipoxygenase D (*LOXD*), allene oxide synthase (*AOS1*), and allene oxide cyclase (*AOC*) and the JA content were noticeably increased at 4 and 8 days of natural water loss (Figures 5C–F). Under well-watered conditions, except for *AOS1* at 4 days, the expression of the other genes showed no significant difference between *MYC2*-OE and M82 plants. However, at 8 days of water loss, the expression of JA synthesis genes in *MYC2*-OE was significantly higher than that in M82 (Figures 5C–E). Accordingly, JA accumulation was higher in *MYC2*-OE plants than in M82 after 8 days of water loss (Figure 5F). Based on these results, the overexpression of *SlMYC2* promoted the accumulation of ABA and JA in response to water loss.

### Overexpression of *SlMYC2* reduced flavonol biosynthesis by inhibiting *SlCHS1* expression

Because elevated flavonol accumulation in guard cells has been confirmed to decrease ROS concentrations in *Arabidopsis* and tomato (Watkins et al., 2014, 2017) and *MYC2* has been suggested to regulate flavonoid synthesis (Yadav et al., 2005; Ogawa et al., 2017), we asked whether *SlMYC2* regulated ROS production by regulating flavonol levels in guard cells during drought stress.

**Figure 6:**
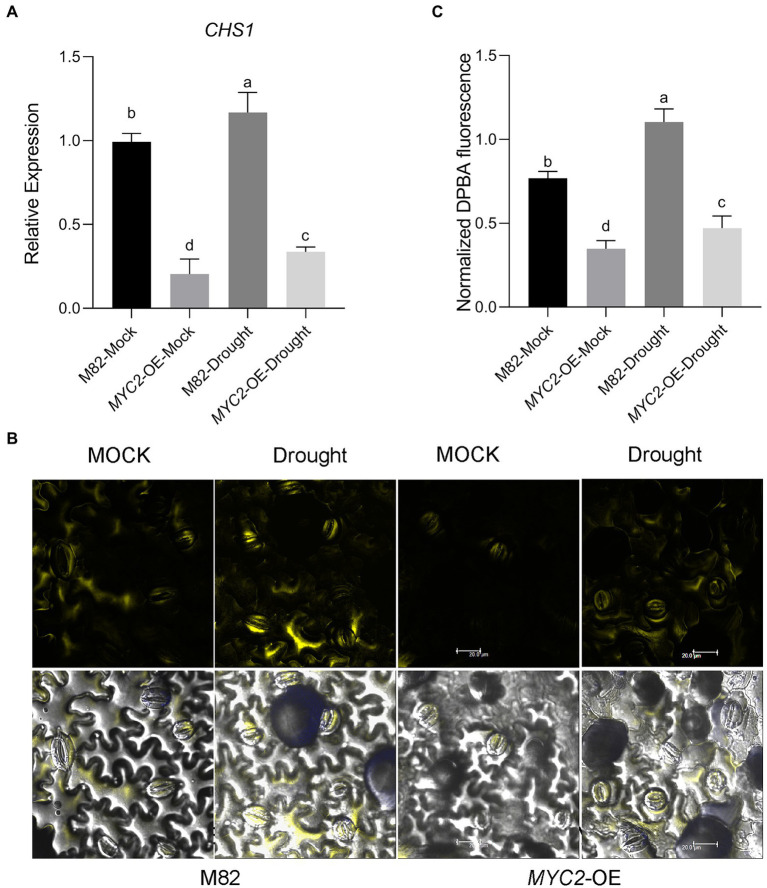
Overexpression of *SlMYC2* decreased the flavonol content by inhibiting the expression of *SlCHS1* in plants under drought stress. **(A)** The expression of *SlCHS1* in tomato guard cells in *MYC2*-OE and M82 plants subjected to drought treatment and mock treatment. **(B)** Confocal micrographs of DPBA-stained guard cells of *MYC2*-OE and M82 plants subjected to drought and mock treatments. DPBA fluorescence is shown in yellow (bar = 20 μm). **(C)** Quantification of DPBA fluorescence in guard cells (M82: *n* = 499; *MYC2*-OE: *n* = 493; M82-Drought: *n* = 498; *MYC2*-OE-Drought: *n* = 505). Data were obtained from three experiments. Different letters represent significant differences (*p* < 0.0001), as determined using Tukey’s multiple comparisons test.

First, we assessed *SlCHS1* mRNA (*Solyc09g091510.2*) expression in guard cells; this gene encodes the chalcone synthase controlling the first step in the flavonoid synthesis pathway. We found that the transcript was significantly upregulated after 8 days of water loss (Figure 6A). Moreover, *SlCHS1* expression was downregulated in *MYC2-*OE plants under both mock and drought stress conditions (Figure 6A). Then, the flavonol content in guard cells was detected using diphenyl boric acid 2-aminoethyl ester (DPBA) staining. Flavonol was located mainly in guard cells and less in pavement cells (Figure 6B). Water loss significantly induced flavonol accumulation in guard cells, and the flavonol content was much lower in *MYC2-*OE than in M82 plants under both water loss and well-watered conditions, consistent with the expression of *SlCHS1* (Figures 6B,C). All the aforementioned data suggested that *SlMYC2* negatively regulated flavonol synthesis by inhibiting *SlCHS1* expression.

### *SlCHS1*-silenced plants exhibit less sensitivity to drought, a decreased stomatal aperture and increased ROS accumulation

**Figure 7:**
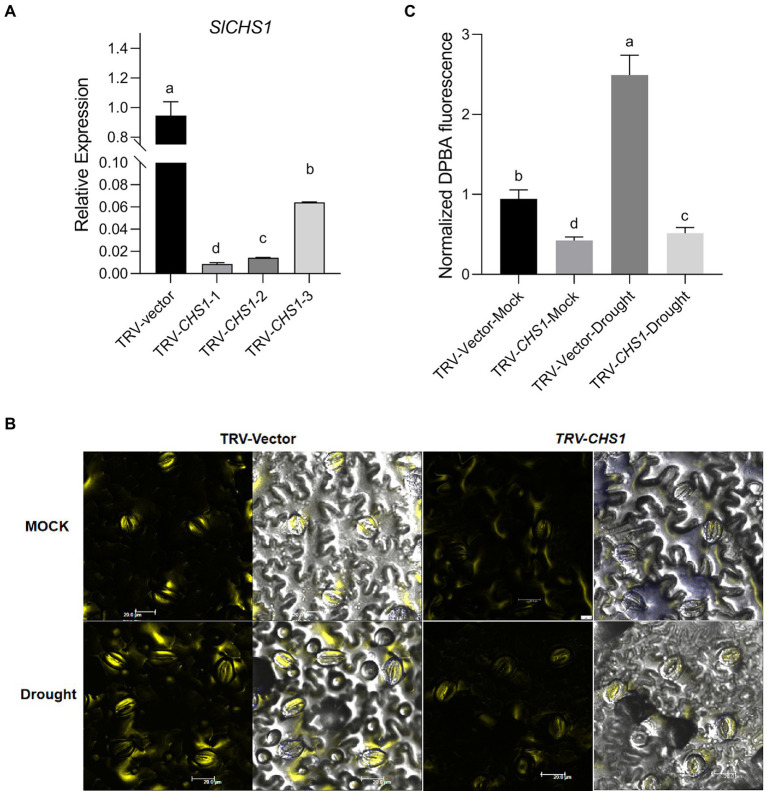
*SlCHS1*-silenced plants exhibited a lower flavonol content in guard cells. **(A)** The expression of *SlCHS1* in leaves of TRV-Vector and TRV-*CHS1* tomato plants. **(B)** Confocal micrographs of DPBA-stained guard cells of TRV-Vector and TRV-*CHS1* plants. DPBA fluorescence is shown in yellow (bar = 20 μm). **(C)** Quantification of DPBA fluorescence in guard cells of TRV-Vector and TRV-*CHS1* plants subjected to 8-day water loss and mock treatments (TRV-vector-MOCK: *n* = 491; TRV-*CHS1*-MOCK: *n* = 510; TRV-vector-Drought: *n* = 494; TRV-*CHS1*-Drought: *n* = 505). Data were obtained from three experiments. Different letters represent significant differences (*p* < 0.0001) determined using Tukey’s multiple comparisons test.

We transiently silenced the *SlCHS1* gene in Castlemart (CM) plants to investigate the role of *SlCHS1* in the response to drought stress. First, we measured the accumulation of *SlCHS1* in leaves using RT–qPCR and found an average 30-fold decrease in *SlCHS1* expression in tobacco rattle virus (TRV)-*CHS1*-expressing plants compared with TRV-Vector-expressing plants (Figure 7A). Subsequently, the flavonol contents of guard cells in TRV-*CHS1*-expressing and TRV-Vector-expressing plants were measured. Because of the partial silencing of *SlCHS1* expression, the flavonol content in guard cells was much level in TRV-*CHS1*-expressing plants than in TRV-Vector-expressing plants, and the drought-induced increase in flavonol was compromised by silencing *SlCHS1* (Figures 7B,C).

**Figure 8:**
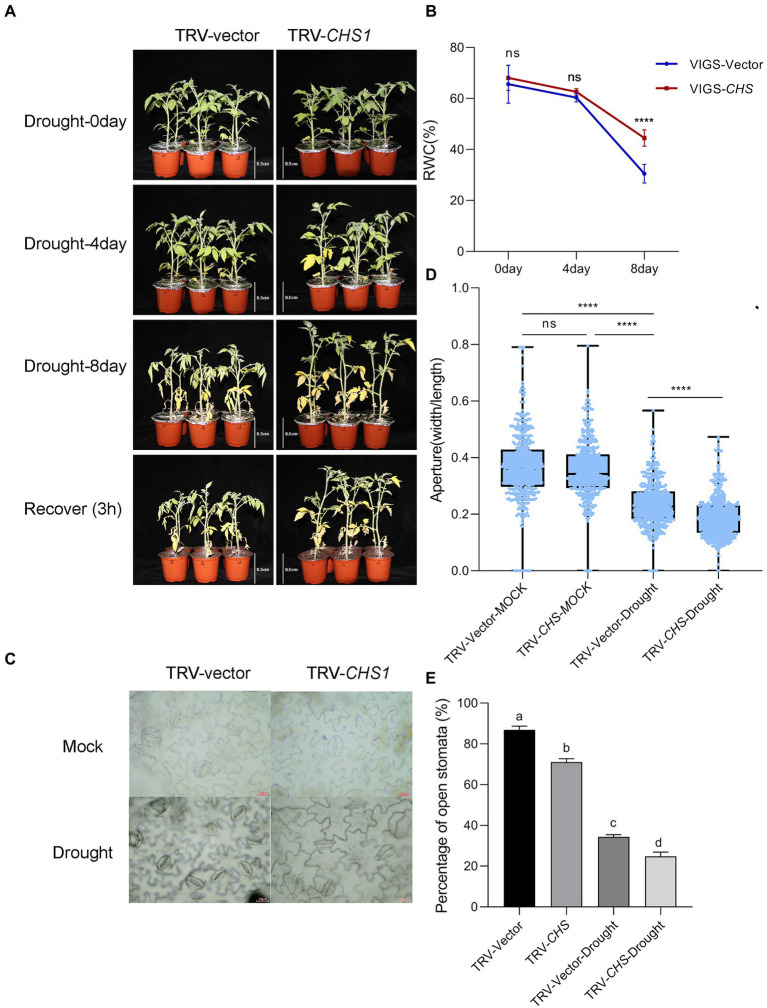
*SlCHS1*-silenced plants were more tolerant to drought. The phenotype **(A)** and RWC **(B)** of TRV-vector and TRV-*CHS1* plants cultivated under 8-day water loss and mock conditions (bar = 8.5 cm). **(C)** Typical images of stomata in TRV-vector and TRV-*CHS1* plants cultivated under 8-day water loss conditions (bar = 20 μm). **(D,E)** Stomatal aperture (*n* = 500) and the percentage of open stomata (170 for each replicate, three replicates, *n* = 510) in TRV-vector and TRV-*CHS1* plants with and without 8 days of water loss. Data were obtained from three experiments. Asterisks represent significant differences (****p < 0.0001), and different letters indicate significant differences (*p* < 0.001) determined using Tukey’s multiple comparisons test.

**Figure 9:**
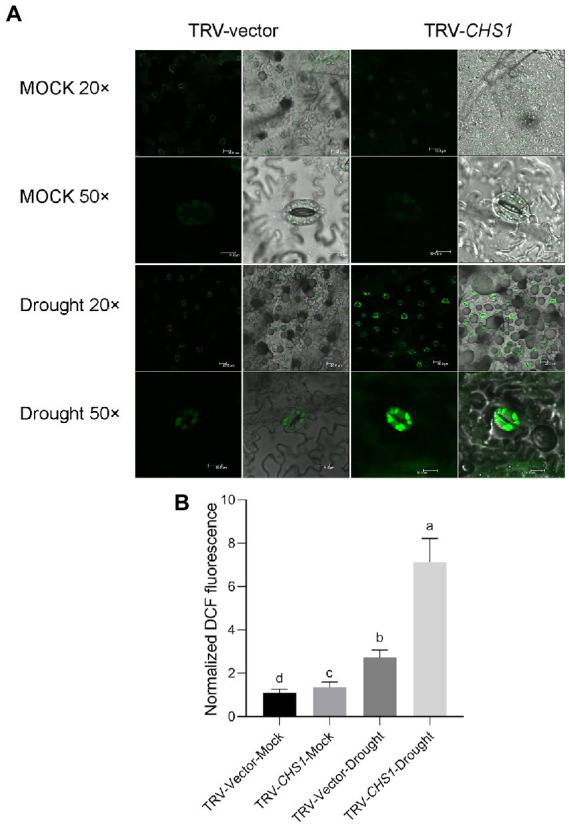
*SlCHS1*-silenced plants exhibited more ROS accumulation in guard cells under drought stress. **(A)** Confocal micrographs of H_2_DCF-DA-stained guard cells of TRV-Vector and TRV-*CHS1* plants subjected to drought and mock treatments. DCF fluorescence is shown in green (bar = 20 μm). **(B)** Quantification of DCF fluorescence in guard cells (TRV-vector-MOCK: *n* = 498; TRV-*CHS1*-MOCK: *n* = 500; TRV-vector-Drought: *n* = 498; TRV-*CHS1*-Drought: *n* = 494). Data were obtained from three experiments. Different letters represent significant differences (*p* < 0.0001), as determined using Tukey’s multiple comparisons test.

The growth of TRV-*CHS1* and TRV-Vector plants was not different under well-watered conditions ( Supplementary Figure 3). Water loss treatment similar to that in *MYC2*-OE plants was conducted in TRV-*CHS1* and TRV-Vector plants. As shown in Figure 8A, the phenotype of TRV-*CHS1* plants under drought stress revealed less sensitivity than that of TRV-Vector plants after 8 days of water loss. The RWC of TRV-*CHS1* plants was significantly higher than that of TRV-vector plants after 8 days of water loss (Figure 8B). Under well-watered conditions, no difference in stomatal aperture was observed between TRV-vector and TRV-*CHS1* plants. Additionally, the stomatal aperture of TRV-*CHS1* plants was smaller, and fewer stomata remained open than in TRV-Vector plants after drought stress (Figures 8C–E). The ROS contents in guard cells of TRV-*CHS1* plants were increased by 2-fold compared with TRV-vector plants under drought stress, although they showed similar ROS levels under well-watered conditions (Figures 9A,B). A strong negative correlation between flavonol and ROS contents was observed in plants under drought stress (Figures 7B, 9B), suggesting that flavonols may reduce ROS levels in guard cells.

### *SlMYC2* directly regulated *SlCHS1* transcription

Based on our abovementioned findings that *SlMYC2* functions in the repression of *SlCHS1* expression in response to drought stress, we speculated that *SlMYC2* directly regulated *SlCHS1* expression. The promoter analysis showed that two MYC2-binding G-box motifs, *CHS1*-G1 (CACGTT) and *CHS1*-G2 (CACATG), were present in the promoter region of *SlCHS1* (Figure 2A), suggesting the possibility that MYC2 bound to the *CHS1* promoter. Yeast one-hybrid (Y1H) studies suggested that SlMYC2 bound to the DNA fragment of the *SlCHS1* promoter containing the G-box motif *CHS1*-G1 (Figure 2B). Transactivation tests using a dual-luciferase system showed that *SlMYC2* expression inactivated the *SlCHS1* promoter (Figures 2C,D). Then, we used *MYC2*-OE plants carrying a green fluorescence protein (GFP) tag to perform chromatin immunosuppression (ChIP)-qPCR and used a GFP antibody to detect whether the MYC2 protein bound to the fragments of the *CHS1* promoter. The DNA fragment (P1) containing the G-box, *CHS1*-G1, was remarkably enriched, while *CHS1*-G2(P4) and three nonbinding sites (P2, P3 and P5) were not enriched (Figures 2A,E). These results suggest that *SlMYC2* directly represses the expression of *SlCHS1*.

## Discussion

**Figure 10:**
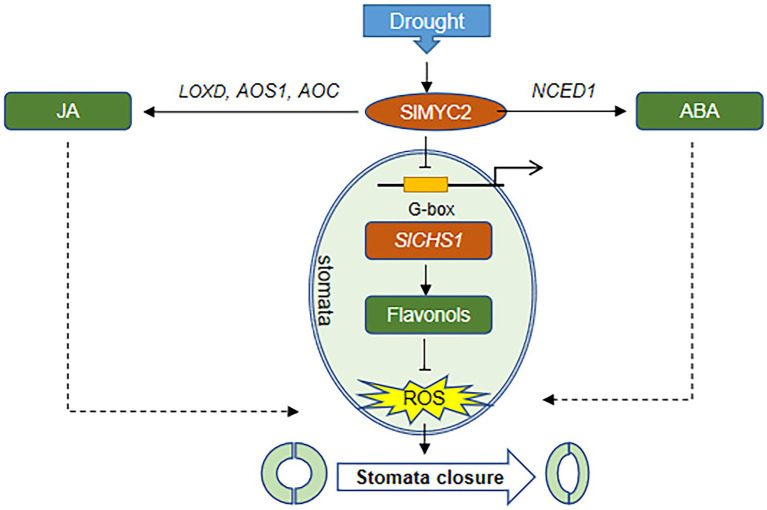
The proposed mechanism by which *SlMYC2* responds to drought stress and regulates stomatal closure. In response to drought stress, *SlMYC2* expression is induced, which promotes the accumulation of JA and ABA by activating the expression of hormone biosynthetic genes. SlMYC2 also interacts with the *SlCHS1* promoter to repress its expression, resulting in a reduced flavonol content and increased ROS levels in guard cells. Solid arrows represent positive regulation proven in this study, and dashed arrows represent positive regulation proven in other studies. Blunt arrows represent negative modulation.

Stomatal closure limits transpiration to maintain the water content in plants under water-deficient conditions (Kim et al., 2010). According to many studies, JA signalling pathways are implicated in drought stress by regulating stomatal closure, and the JA content is clearly increased in response to drought (Kim et al., 2017; Ruan et al., 2019). The biosynthesis of JA begins with the release of *α*-linolenic acid from chloroplast membranes by phospholipase A_1_. Then, α-linolenic acid is catalysed to 12-oxo-phytodienoic acid by 13-LOX, AOS, and AOC (Wasternack and Hause, 2013). In this study, the transcript levels of the JA synthesis genes *AOC, AOS1* and *LOXD* and JA content increased in response to natural water loss (Figures 5C–F, 10).

Additionally, MYC2, the JA signalling “Master Switch” (Breeze, 2019), was proven to function as a positive regulator of JA signalling in the drought stress response (Seo et al., 2011; Li et al., 2019). We found that *SlMYC2* was involved in the drought stress response, and overexpression of *SlMYC2* resulted in reduced sensitivity to drought, a decreased stomatal aperture and increased ROS accumulation in guard cells (Figures 1–4, 10), suggesting that *SlMYC2* is closely related to drought stress resistance. Furthermore, we also confirmed the role of *SlMYC2* in regulating JA accumulation in plants under drought stress. Overexpression of *SlMYC2* facilitated drought-induced JA production by increasing the expression of *LOXD, AOS1* and *AOC* (Figures 5C–F, 10). In fact, several studies have shown that JA biosynthesis is regulated by feedback from *MYC2* (Dombrecht et al., 2007; Du et al., 2017), and MYC2 directly binds to the promoters of several JA biosynthetic genes, such as *LOX2/3/4*, *AOC3*, *AOS*, and *OPR3* in *Arabidopsis*, which increases the expression levels of these genes (Hou et al., 2010; Zhang et al., 2020).

MYC2 also plays a vital role in the crosstalk between JA and ABA (Kazan and Manners, 2008; Du et al., 2017). For example, MYC2 is implicated in ABA-modulated gene expression in *Arabidopsis* (Abe et al., 2003; Kazan and Manners, 2008). Seed germination studies in *Arabidopsis* showed that MYC2 plays an active role in ABA biosynthesis and the expression of responsive genes (Verma et al., 2020). In this study, SlMYC2 regulated the transcription of *NCED1*, which is a guard cell-specific gene and encodes a rate-limiting enzyme in the ABA biosynthesis pathway in tomato (Du et al., 2014), resulting in an increase in the ABA content in plants under drought stress (Figures 5A,B, 10), which may be one reason why *SlMYC2* increases stomatal closure in response to water loss.

Interestingly, recent studies have shown that the JA synthesis-related gene *CmLOX10* in oriental melon and the JA signalling-related gene *OsJAZ1* in rice respond to drought stress (Fu et al., 2017; Xing et al., 2020). *CmLOX10* enhanced plant drought tolerance and promoted stomatal closure by increasing the biosynthesis of JA rather than ABA, and CmMYC2 directly bound to the *CmLOX10* promoter (Xing et al., 2020). In contrast, *OsJAZ1* negatively modulates drought resistance of rice partially *via* the ABA and JA synthesis pathways (Fu et al., 2017). The JAZ protein interacts with and inhibits MYC2 function in the JA signalling pathway (Thines et al., 2007). Combined with our result (Figures 5, 10) that *SlMYC2* increased JA and ABA accumulation during drought tolerance, we proposed that JA biosynthesis is regulated by feedback through *SlMYC2* in tomato and that *SlMYC2* plays a central role in the crosstalk of JA and ABA during drought-induced stomatal movement in tomato.

ROS activate multiple signalling pathways in different organisms and have been recognized as core components of the complex signalling network in plants (Mittler et al., 2011). During stomatal closure signalling, a rapid ROS burst resulting from a shift in the balance between scavenging and the production rate generates a signal (Mittler et al., 2004; Vanderauwera et al., 2011). Nevertheless, ROS signalling must be strictly regulated by protein and/or small-molecule antioxidants to prevent oxidative damage (Apel and Hirt, 2004; Nadarajah, 2020), a process mediated by ROS scavengers, including flavonoids and glutathione (Munemasa et al., 2013; Watkins et al., 2017). In our study, overexpression of *SlMYC2* increased the ROS content in guard cells, and we also found that *SlMYC2* regulated ROS levels in guard cells, probably by regulating flavonol synthesis in plants under drought stress (Figures 4, 6, 10), as reflected by the observation that *SlCHS1* expression and the flavonol content were both reduced in *MYC2-*OE plants (Figure 6). Furthermore, by performing ChIP–qPCR, Y1H and dual-luciferase reporter gene assays, we showed that *SlCHS1* is a direct target and is repressed by *SlMYC2* (Figures 2, 10). In addition, VIGS-*SlCHS1* plants phenocopied *Sl**MYC2-*OE plants in terms of drought resistance, stomatal closure, flavonol and ROS levels (Figures 7–9). In addition, ABA and JA also increased ROS levels in guard cells, which act as secondary messengers of ABA and JA signalling (Figure 10; Daszkowska-Golec and Szarejko, 2013; Simontacchi et al., 2013). In our research, *SlMYC2* overexpression increased JA and ABA accumulation during drought tolerance; thus, this result may indicate another pathway by which *SlMYC2* increases the ROS level (Figure 10). Taken together, these results suggest that the *SlMYC2* gene finely tunes ROS homeostasis at multiple levels to enhance drought resistance in tomato (Figure 10).

Previous studies have reported controversial results for the role of *MYC2* in regulating *CHS* (Yadav et al., 2005; Ogawa et al., 2017), and less is known about how *MYC2* regulates *CHS* under drought stress. Our study indicates that *SlMYC2* functions as a negative regulator of *SlCHS1* expression and leads to the reduced accumulation of flavonols, and the latter inhibit ROS-driven stomatal closure. We also observed increases in *SlCHS1* expression and the flavonol content during water loss (Figures 6, 7). Meanwhile, a negative correlation between the flavonol content and ROS level was observed during drought stress (Figures 4, 6, 7, 9). These findings imply that the flavonol-ROS balance plays a critical role in regulating stomatal dynamics under stress conditions. Taken together, plants integrate JA, ABA, flavonol and ROS signals through the modulation of MYC2 during drought-mediated stomatal movement (Figure 10), which will help to dissect the mechanism underlying the integration of these signals in future studies. Although our results suggested that *SlMYC2* was involved in regulating drought-induced JA and ABA biosynthesis, investigations examining whether *SlMYC2* directly interacts with members of the JA and ABA synthetic pathways, regulates genes in the ABA signalling pathway, or mediates ROS production in guard cells are worthwhile.

## Data availability statement

The original contributions presented in the study are included in the article/ Supplementary Material, further inquiries can be directed to the corresponding authors.

## Author contributions

Z-WC and W-SH conceived and designed the study. X-BQ and W-JJ performed the experiments. X-BQ wrote the paper. Z-WC and W-SH edited and reviewed the paper. All authors read and approved the manuscript.

## Funding

This work was funded by grants from the National Key R&D Program of China (2019YFD1000300), the Beijing Natural Science Foundation project – Key Project of Science and Technology Plan of Beijing Education Commission (KZ202010020027), Innovation Program of Beijing Academy of Agricultural and Forestry Sciences (KJCX20220406), and the Beijing Innovation Consortium of Agriculture Research System (BAIC01-2021).

## Conflict of interest

X-BQ is employed by Bei Jing Bei Nong Enterprise Management Co., Ltd.

The remaining authors declare that the research was conducted in the absence of any commercial or financial relationships that could be construed as a potential conflict of interest.

## Publisher’s note

All claims expressed in this article are solely those of the authors and do not necessarily represent those of their affiliated organizations, or those of the publisher, the editors and the reviewers. Any product that may be evaluated in this article, or claim that may be made by its manufacturer, is not guaranteed or endorsed by the publisher.
