# Supplementary figures and images for: SlMYC2 mediates stomatal movement in response to drought stress by repressing SlCHS1 expression
Source: Front Plant Sci. 2022 Jul 22;13:952758. doi: 10.3389/fpls.2022.952758 (PMC9354244; doi:10.3389/fpls.2022.952758)

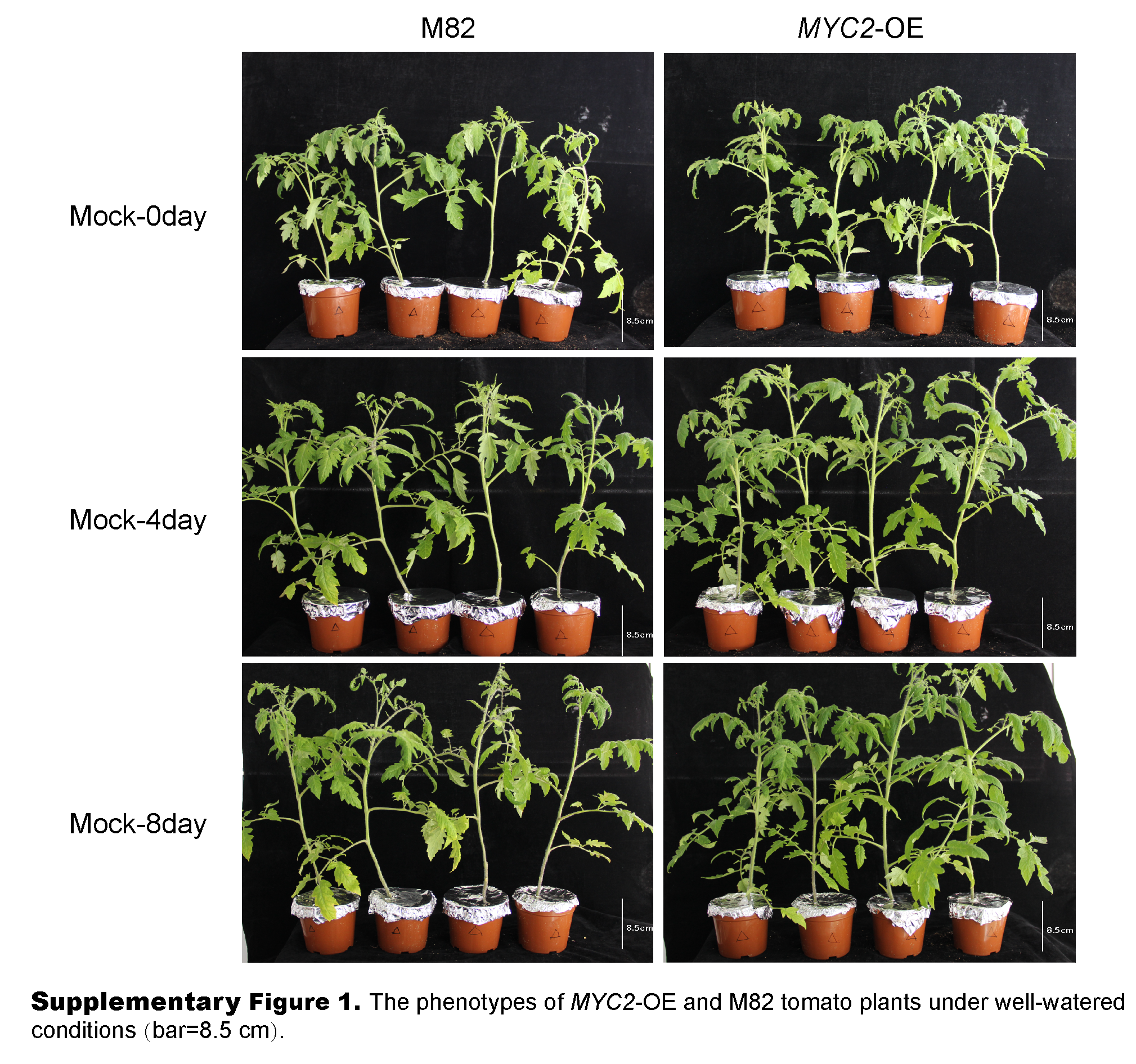

Supplement: Supplementary file 1 [file Image_1.TIF]

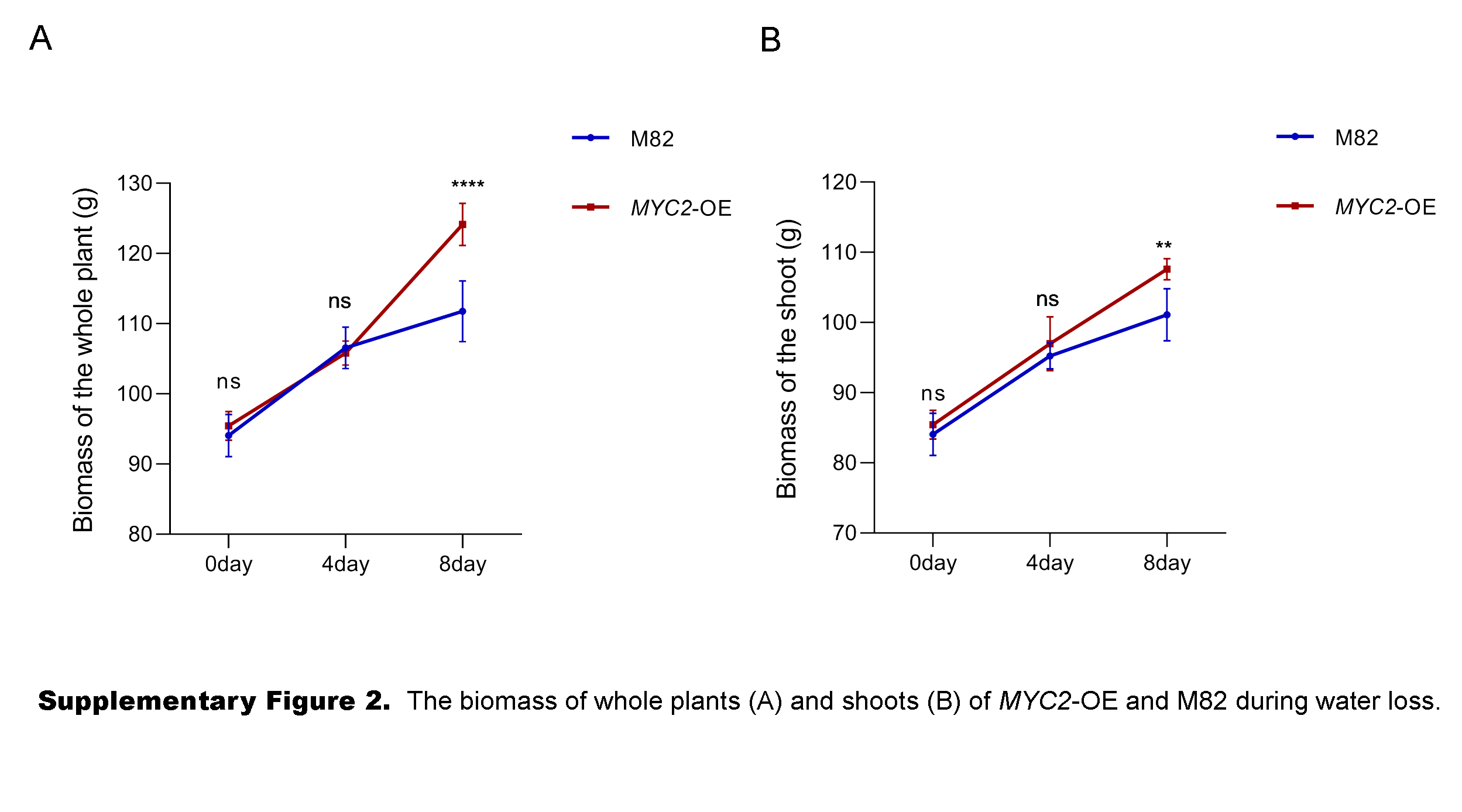

Supplement: Supplementary file 2 [file Image_2.TIF]

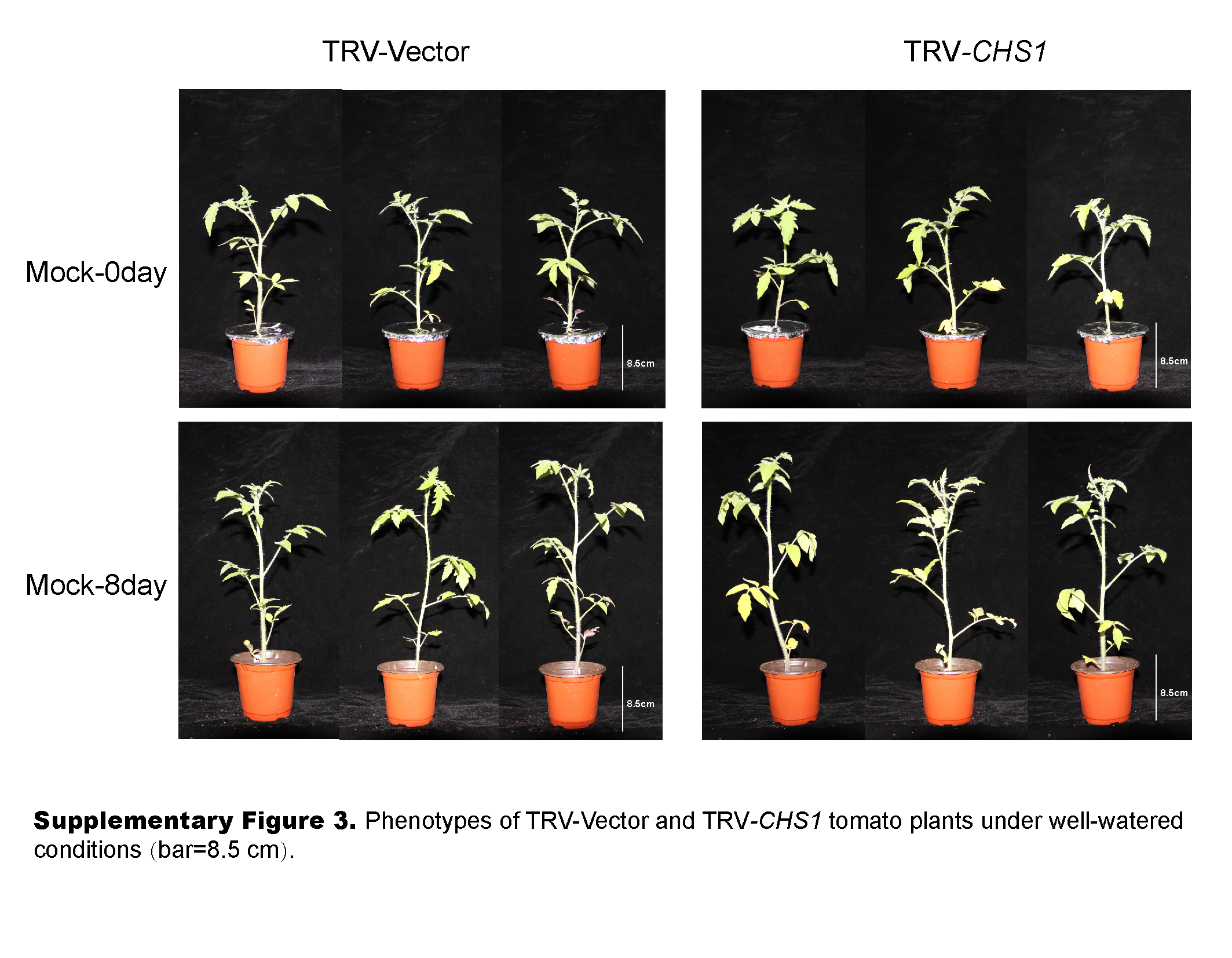

Supplement: Supplementary file 3 [file Image_3.TIF]
