# Supplementary material for: SlMYC2 mediates stomatal movement in response to drought stress by repressing SlCHS1 expression
Source: Front Plant Sci. 2022 Jul 22;13:952758. doi: 10.3389/fpls.2022.952758 (PMC9354244; doi:10.3389/fpls.2022.952758)
Supplement: Supplementary file 4 [file Table_1.DOCX]

**Supplementary Table S1.** The primer sequences used in this study.

Quantitative RT-PCR:

| **Gene** | **Forward primer 5’ - 3’** | **Reverse primer 5’ - 3’** |
| --- | --- | --- |
| *Actin2* | TTGCTGACCGTATGAGCAAG | GGACAATGGATGGACCAGAC |
| *CHS1* | CCCAATTATAGGGGTCGAAAGA | GGAACATCCTTGAGTAAGTGGA |
| *MYC2* | ATCTGCATCTACTTTATCCGCA | GACGATTGCCAAAAGATAGCAT |
| *NCED1* | CAGAACTGCAAATTGTTAACGC | GGCGTTTATGAATGTTCCATGA |
| *LoxD* | TCCATAACTTAATTCCATCTC | TGATAGTGCTAACAACCT |
| *AOS1* | CATCATCATCGTCATCAC | GAAGTAATCAAGTCTGTCTT |
| *AOC* | CTATCTTCTGCCTTCCAA | TGTTAGTTGAATCTGTTGAG |

ChIP-qPCR:

| **Flagments of**  ***CHS1* promoter** | **Forward primer 5’ - 3’** | **Reverse primer 5’ - 3’** |
| --- | --- | --- |
| *CHS1*-P1 | ACATTCTCCTAATGGAACCA | GGAACATCCTTGAGTAAGTGGA |
| *CHS1*-P2 | CGAAGTTGAGGTATATCAGT | TTCATAAGGTAGGAGCAAGA |
| *CHS1*-P3 | TGCTCCTACCTTATGAACC | TTGGACAAGAAGTTGACAC |
| *CHS1*-P4 | TCTATCACTTTTGCTAAT | TAGGAAAACAATGAATGC |
| *CHS1*-P5 | AAGTAGGTAGCTGTCCAATA | CGTGCTTCAACAACTAATG |

Y1H assay:

| **G-box motifs of *CHS1* promoter** | **Forward primer 5’ - 3’** | **Reverse primer 5’ - 3’** |
| --- | --- | --- |
| G1 | CGTTATACCCTTGTTTTC | ATTTCTTTGGTTTGGTTC |
| G2 | AAGAATATTACGGTTCGA | CATAAGGTAGGAGCAAGA |
